# Cardiac magnetic resonance myocardial feature-tracking: the effect of treatment in patients with adult-onset growth hormone deficiency and acromegaly

**DOI:** 10.1186/1532-429X-15-S1-E55

**Published:** 2013-01-30

**Authors:** Mahvesh R Javaid, Ian S Stone, Ashley B Grossman, Marta Korbonits, Julia D Thomas, Steffen E Petersen, Ceri Davies

**Affiliations:** 1 Cardiovascular Biomedical Research Unit, Barts and the London School of Medicine and Dentistry, Queen Mary, University of London, London, UK; 2 Oxford Centre for Diabetes, Endocrinology and Metabolism, University of Oxford, Oxford, UK; 3 Centre for Endocrinology, William Harvey Research Institute, Barts and the London School of Medicine and Dentistry, Queen Mary, University of London, London, UK

## Background

We have previously documented abnormalities in cardiac structure in patients with adult-onset growth hormone deficiency (GHD) and acromegaly which partially resolved during treatment (Dattani *et al.* 2012, Zemrak *et al. *2012).

No treatment effect was seen on global measures of cardiac function e.g. left ventricular ejection fraction.

The aim of this study was to use quantitative strain parameters from cardiac magnetic resonance (CMR) myocardial feature tracking (FT) to assess regional changes in function before and after treatment in patients with GHD and acromegaly.

## Methods

10 patients with GHD, 13 patients with acromegaly and 23 age- and sex-matched normal controls underwent CMR. Patients underwent scanning before and again 12 months after treatment. Radial and circumferential strain parameters were derived from 2-chamber (2CH), 4-chamber (4CH) and basal, mid and apical short axis cine-images using dedicated FT software (Diogenes MRI, TomTec Imaging Systems, Munich, Germany). Comparisons between groups were performed using the T-test.

## Results

There were no significant differences in any FT derived parameters between normal controls and patients with GHD.

There was a significant difference in 4CH-circumferential strain in patients with acromegaly compared to normal controls (-7.6% vs -5.0%, p=0.01). There were no significant differences in the remaining parameters.

The effect of treatment on FT derived indices is shown in Tables 1 and 2.

**Table 1:** The effect on feature-tracking parameters before and after one year of treatment in acromegaly patients.

Feature-tracking Parameters	2C Pre	2C Post	4C Pre	4C Post	Basal Pre	Basal Post	Mid Pre	Mid Post	Apex Pre	Apex Post
Radial Strain (%)	16.6 ± 6.9	14.9 ± 5.2	13.1 ± 6.3	15.1 ± 3.9	22.2 ± 14.4	20.0 ± 5.7	18.7 ± 7.7	16.1 ± 6.0	17.1 ± 5.8	19.5 ± 15.9

Circumferential Strain (%)	-7.17 ± 2.8	-7.23 ± 2.4	-7.55 ± 1.9	-5.69 ± 2.0	-9.87 ± 2.3	-10.4 ± 1.6	-9.19 ± 3.0	-8.86 ± 2.3	-9.99 ± 2.7	-10.6 ± 3.4

The values represent mean ± 1SD Abbreviation: 2C - 2 Chambers, 4C - 4 Chambers, Pre - Pretreatment, Post - Post-treament

**Table 2:** The effect on feature-tracking parameters before and after one year of treatment in GHD patients.

Feature-tracking Parameters	2C Pre	2C Post	4C Pre	4C Post	Basal Pre	Basal Post	Mid Pre	Mid Post	Apex Pre	Apex Post
Radial Strain (%)	16.3 ± 5.8	19.4 ± 5.2	14.8 ± 5.2	14.0 ± 6.5	20.5 ± 8.1	19.4 ± 7.5	17.0 ± 5.4	15.1 ± 2.2	19.0 ± 9.0	16.1 ± 8.6

Circumferential Strain (%)	-7.83 ± 1.7	-7.59 ± 2.05	-6.07 ± 3.4	-4.72 ± 5.3	-10.7 ± 2.6	-10.2 ± 2.6	-8.43 ± 4.2	-9.25 ± 2.5	-9.62 ± 2.7	-8.84 ± 3.3

The values represent mean ± 1SD Abbreviation: 2C - 2 Chambers, 4C - 4 Chambers, Pre - Pretreatment, Post - Post-treatment

## Conclusions

Other than 4-CH circumferential strain, no differences were seen in any FT derived strain measurements between patients with GHD and normal controls, patients with acromegaly and normal controls and following 12 months of treatment.

The structural changes seen in patients with acromegaly and GHD deficiency may, therefore, precede significant functional abnormalities. Early identification and treatment of these patients may prevent the development of functionally important myocardial damage.

## Funding

This study has been supported by departmental grants from NIHR, Pfizer and Novartis.

